# Nitrile hydratase of *Rhodococcus erythropolis*: characterization of the enzyme and the use of whole cells for biotransformation of nitriles

**DOI:** 10.1007/s13205-012-0104-2

**Published:** 2012-12-16

**Authors:** Ashwini L. Kamble, Linga Banoth, Vachan Singh Meena, Amit Singh, Yusuf Chisti, U. C. Banerjee

**Affiliations:** 1 Department of Pharmaceutical Technology (Biotechnology), National Institute of Pharmaceutical Education and Research, Sector-67, S.A.S. Nagar, Mohali, 160062 Punjab India; 2 School of Engineering, Massey University, Private Bag 11 222, Palmerston North, New Zealand

**Keywords:** Nitrile hydratase, *Rhodococcuserythropolis*, Biotransformations, Nitriles, Amides

## Abstract

The intracellular cobalt-type nitrile hydratase was purified from the bacterium *Rhodococcuserythropolis*. The pure enzyme consisted of two subunits of 29 and 30 kDa. The molecular weight of the native enzyme was estimated to be 65 kDa. At 25 °C the enzyme had a half-life of 25 h. The Michaelis–Menten constants *K*_m_ and *v*_max_ for the enzyme were 0.624 mM and 5.12 μmol/min/mg, respectively, using 3-cyanopyridine as the substrate. The enzyme-containing freely-suspended bacterial cells and the cells immobilized within alginate beads were evaluated for converting the various nitriles to amides. In a packed bed reactor, alginate beads (2 % alginate; 3 mm bead diameter) containing 200 mg/mL of cells, achieved a conversion of >90 % for benzonitrile and 4-cyanopyridine in 38 h (25 °C, pH 7.0) at a feed substrate concentration of 100 mM. The beads could be reused for up to six reaction cycles.

## Introduction

Nitrile hydratases (EC 4.2.1.84) are metaloenzymes that contain either non-heme iron(III) or non-corrinoid cobalt(III) at the active site (Sugiura et al. 1987; Payne et al. 1997; Yamada et al. 2001; Mascharak 2002). A nitrile hydratase is composed of two subunits, α and β that differ in the amino acid sequence (Mascharak 2002). These enzymes catalyze the hydration of nitriles to amides (Payne et al. 1997; Mylerova and Martinkova 2003). This reaction is of interest in various synthetic and bioremediation applications (Mylerova and Martinkova 2003; Sachi and Lindsay 2007). Nitrile hydratase from *Rhodococcus rhodochrous* is used for the industrial production of acrylamide from acrylonitrile (Kobayashi and Shimizu 2000; Raj et al. 2006). Similarly, a process for the degradation of acetonitrile in wastewater has been described (Hjort et al. 1990). This process uses the nitrile hydratase producing bacterium *Rhodococcus pyridinivorans* (Hjort et al. 1990). Nitrile hydratase has other potential applications, (Mylerova and Martinkova 2003) as various amides are used as pharmaceuticals and agrochemicals. For example, nicotinamide is used as a vitamin B3 (Lin et al. 2001) and pyrazinamide and isoniazid (isonicotinic acid hydrazide) are used as tuberculosis drugs (Ray et al. 2004). Herbicides, such as diflubenzuron, are synthesized from amides (Clifford et al. 1993). In addition, the intracellular nitrile hydratase of *Rhodococcus erythropolis* MTCC 1526 is purified and characterized for possible use as a soluble biocatalyst. Nitrile hydratases from *Pseudomonas chlororaphis* B23 (Nagasawa et al. 1987), *Brevibacterium* R312 (Nagasawa et al. 1986), *Rhodococcus* sp. RHA1 (Sachi and Lindsay 2007) and *R. rhodochrous* PA-34 (Prasad et al. 2009) have been previously characterized.

Nitrile hydratase-mediated hydrolysis of nitriles for the production of high value amides or acids can potentially displace chemical processes with environment-friendly low-temperature processes that operate at near physiological pH (Brady et al. 2004; Cantarella et al. 2006). In view of their high selectivity and specificity, the enzyme-catalyzed bioconversions occur with few or no side reactions. If used in an immobilized form, enzymes and cells can be reused repeatedly as biocatalysts (Nigam et al. 2009).

This work is focused on the use of nitrile hydratase containing whole cells of the *R. erythropolis* MTCC 1526 as an immobilized biocatalyst for the transformation of various nitriles (3-cyanopyridine, 4-cyanopyridine, benzonitrile, pyrazinonitrile, isobutyronitrile) to amides.

## Materials and methods

### Chemicals

Standard nitriles, amides, tetramethylethylenediamine (TEMED), ammonium persulfate, acrylamide and bis-acrylamide were purchased from Sigma-Aldrich (Germany). Growth media components were purchased from Hi-Media Inc. (Mumbai, India). Matrices for protein purification were sourced from Amersham Biosciences AB (Uppsala, Sweden). Solvents for HPLC were procured from Mallinckrodt Baker Inc. (Phillipsburg, USA) and Ranbaxy Chemicals Co. (Mohali, India). All chemicals and reagents were of analytical grade.

### Microorganism and culture conditions

*Rhodococcus erythropolis* MTCC 1526 (procured from Microbial Type Culture Collection, Institute of Microbial Technology, Chandigarh, India) was maintained on a nutrient agar medium (pH 7.0) as described by Tanaka and Kimura (1972). Seed culture was prepared by inoculating a single colony of the bacterium in 20 mL of a nutrient medium contained in a 100 mL Erlenmeyer flask. The nutrient medium was prepared by dissolving the following components in deionized water (g/L): peptone 5, beef extract 1.5, yeast extract 1.5 and NaCl 5. The seed culture was incubated at 25 °C for 24 h on a rotary shaker at 200 rpm. The inoculum was transferred to 500 mL Erlenmeyer flask containing 100 mL of the production medium (initial pH 8.0) and grown for 60 h at 25 °C. The production medium contained the following in deionized water (g/L): peptone 5, beef extract 1.5, yeast extract 1.5, NaCl 5, KH_2_PO_4_ 0.5, K_2_HPO_4_ 0.5, MgSO_4_ 0.5, glycerol 10 and CoCl_2_ 0.01.

### Nitrile hydratase activity assay

Nitrile hydratase activity was measured using a modification of the method of Nagasawa et al. (1986). Thus, 100 mg/mL of cell mass (wet weight), or the cell-free culture supernatant, was mixed with phosphate buffer (10 mM, pH 7.0) containing 400 μL of 5 mM nitriles. The reaction was allowed to proceed for 60 min at 20 °C for whole cells, or for 30 min in the case of the cell-free extract. The reaction flask was continuously mixed at 160 rpm. The reaction was stopped by adding 0.2 mL of 1 M HCl. Cells were removed by centrifugation at 13,000*g* for 10 min. The amount of nicotinamide formed in the reaction mixture was determined by HPLC.

### Analytical methods

#### Reversed phase HPLC

The different nitriles and amides were detected by high performance liquid chromatography (Shimadzu 10AD VP, Kyoto, Japan) using a reversed phase column (4.6 × 250 mm; Phenomenex, USA). For the detection of benzonitrile, benzamide, isobutyronitrile, isobutyramide, pyrazinecarbonitrile and pyrazinecarboxamide, a mobile phase consisting of acetonitrile:water 65:35 v/v with a flow rate of 0.5 mL/min was used. Aromatic nitriles and the corresponding amides were detected at 254 nm while the aliphatic nitriles and their corresponding amides were detected at 210 nm. In the case of 3-cyanopyridine, nicotinamide, 4-cyanopyridine and isonicotinamide, the mobile phase consisted of phosphate buffer (50 mM, pH 7.0) and acetonitrile mixed in the proportion of 95:5 by volume. The flow rate was 1 mL/min and detection was at 230 nm.

#### Protein content

Protein concentration in the various samples was estimated by Bradford’s dye binding assay using bovine serum albumin as the standard (Bradford 1976). Absorbance was monitored at 595 nm in a microplate scanning spectrophotometer (E max, Molecular Devices, Precision Microplate Reader, USA).

#### Enzyme purification

Protein purification was performed using a fast performance liquid chromatography (FPLC) system (Akta Prime, Amersham Pharmacia Biotech, Uppsala, Sweden). All process was carried out at 4 °C in buffer A (50 mM phosphate buffer, pH 6.8, containing 20 mM sodium butyrate). The nitrile hydratase activity in various fractions was determined using the earlier specified method.

#### Preparation of cell-free extract

*Rhodococcus erythropolis* cells were harvested by centrifugation (7,000*g*, 15 min) and washed with the earlier specified buffer A. The washed cell mass was suspended in 200 mL of buffer A and then disrupted by ultrasonication for 20 min at 4 °C. The cell debris was removed by centrifugation (30,000*g*, 30 min, 4 °C). The clear supernatant thus obtained was used as the cell-free extract in further work.

#### Ammonium sulfate precipitation

Proteins were precipitated by adding ammonium sulfate to the cell-free extract to a level of 30 % of saturation. The mixture was stirred on ice for 1 h. The precipitate formed was removed by centrifugation (30,000*g*, 30 min, 4 °C) and discarded. The clear supernatant obtained was subjected to a further ammonium sulfate precipitation at 60 % of the saturation concentration. The mixture was stirred as before. The precipitate was collected by centrifugation (30,000*g*, 30 min, 4 °C) and dissolved in buffer A. This solution was dialyzed against the same buffer to remove ammonium sulfate.

#### Anion exchange chromatography

The dialyzed solution was applied to a Q-Sepharose column (1.6 × 10 cm) pre-equilibrated with buffer A at a flow rate of 1 mL/min. The enzyme was eluted by NaCl (0–1 M) in buffer A. The active fractions were eluted at 350 mM NaCl concentration. These were pooled and then concentrated with a Centricon YM 10 ultrafiltration membrane (Millipore, Bedford, USA).

#### Hydrophobic interaction chromatography

The concentrated fraction from the previous step was subjected to hydrophobic interaction chromatography. Thus, the concentrated fraction was applied to a phenyl-Sepharose column (1.6 × 15 cm) (Amersham Biosciences, Uppsala, Sweden), pre-equilibrated with 1 M ammonium sulfate in phosphate buffer (50 mM, pH 7.0) and eluted at a flow rate of 1 mL/min using a 1–0 M linear gradient of ammonium sulfate in buffer A. Nitrile hydratase was eluted at 560–450 mM ammonium sulfate concentration.

#### Estimation of the molecular weight of the purified native nitrile hydratase

The molecular weight of the native enzyme was determined using Sephacryl S-200 gel permeation column (700 × 15 mm, 47 μm) (Amersham Biosciences, Uppsala, Sweden). The column was eluted with phosphate buffer (50 mM, pH 6.8) containing 20 mM sodium *n*-butyrate. The mobile phase flow rate was 0.5 mL/min. The column was calibrated with the following standard proteins: thyroglobulin (663 kDa), apoferritin (443 kDa), alcohol dehydrogenase (150 kDa), bovine serum albumin (66 kDa) and carbonic anhydrase (29 kDa).

#### Sodium dodecyl sulfate-polyacrylamide gel electrophoresis (SDS-PAGE)

Denaturing polyacrylamide gel electrophoresis was performed on purified nitrile hydratase using the method described by Laemmeli (1970). A 12 % SDS-PAGE gel was used with tris–glycine buffer system. Gels were developed using a Hoefer miniVE vertical gel electrophoresis system (Amersham Biosciences, Uppsala, Sweden) equipped with a EPS-301 constant power supply. The protein bands were visualized by silver staining (Dráber 1991). The molecular weights of the purified nitrile hydratase subunits (denaturing gel) were estimated by comparison with the migration bands of the standard proteins (Amersham Biosciences, Uppsala, Sweden).

### Characterization of the purified nitrile hydratase

The activity profiles of the purified nitrile hydratase were characterized at various pH and temperature values. The pH stability and thermostability of nitrile hydratase were also determined. The effect of various metal ions and inhibitors on the activity of the enzyme was examined. Substrate specificity was checked using various nitriles. The Michaelis–Menten kinetic parameters *K*_m_ and *v*_max_ were estimated using the Lineweaver–Burk plot method.

### Immobilization of whole cells in alginate

The bacterial cells were recovered from the culture broth by centrifugation (7,000*g*, 15 min), washed with phosphate buffer (10 mM, pH 7.0) and recovered (10,000*g*, 10 min). The washed cells were resuspended in 10 mL of Tris–HCl buffer (20 mM, pH 7.0) to achieve a cell concentration of 200 mg/mL. Prior to mixing with the cells, various amounts of sodium alginate had been dissolved in the Tris–HCl buffer (20 mM, pH 7.0) solution by heating in a microwave oven. This solution was cooled to room temperature and then mixed with the cells. The alginate beads were formed by dripping the resulting cell slurry through a syringe, into a chilled solution of calcium chloride (0.2 mM). The beads were hardened by refrigeration (4 °C) for 30 min and then washed with 0.9 % NaCl.

### Optimization of reaction conditions using immobilized cells

The effect of alginate beads prepared with different concentrations of sodium alginate (1.0–2.5 %, w/v) and bead size were assessed for nitrile conversion. The bead size was controlled by using syringes with different orifice sizes (1–5 mm). The optimal buffer strength for the reaction was identified by carrying out the reaction at various concentrations (20, 50, 100, 150 and 200 mM) of Tris–HCl buffer (pH 7.0). The effect of different substrate concentrations (50–100 mM) on the conversion of 3-cyanopyridine was examined using alginate immobilized cells. The effect of varying cell concentrations (200, 250, 300, 350, 400 and 450 mg/mL) in the immobilized matrix were also observed. The stability and reusability of the alginate beads were improved by strengthening them through a cross linking treatment. For this, after the beads were prepared as previously explained, they were suspended for 1 h in a solution of glutaraldehyde (0.2 M) and polyethyleneimine (0.5 %). Afterwards, the beads were washed thoroughly with deionized water and used for the biotransformation reaction. Samples were collected at regular intervals and analyzed using HPLC.

## Results and discussion

### Enzyme purification

The purification of nitrile hydratase has been reported from certain bacteria (Hjort et al. 1990; Prepechalová et al. 2001). The intracellular nitrile hydratase was purified for a detailed characterization. A 3-step purification process subjected the debris-free homogenate of the disrupted cells to ammonium sulfate fractionation, Q-Sepharose ion exchange chromatography and phenyl-Sepharose hydrophobic interaction chromatography. The protein concentration, enzyme activity, the specific enzyme activity, recovery yield and the purification factor at various stages of the purification process are shown in Table 1.

**Table 1:** Purification of intracellular nitrile hydratase from *R. erythropolis*

Purification step	Total protein (mg)	Total enzyme activity (U)	Total specific activity (U/mg)	Yield (%)	Purification factor
Cell-free extract	1,080	12.5	0.012	100	1.0
Ammonium sulfate precipitation	520	8.8	0.017	70	1.4
Q-Sepharose column	220	6.0	0.027	48	2.3
Phenyl-Sepharose	16	2.2	0.130	17.6	10.8

Ammonium sulfate precipitation removed a lot of unwanted proteins, and 70 % of the initial enzyme activity of the crude extract was recovered from the final precipitate (Table 1). The purification factor at this stage was 1.4 (Table 1). At the final step of purification, the purification factor was nearly 11 and about 18 % of the total initial activity was recovered (Table 1).

### Characterization of the purified nitrile hydratase

#### Molecular weight

**Fig. 1:**
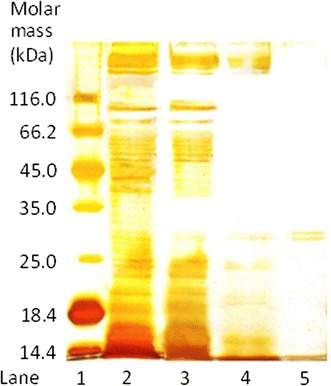
SDS-PAGE of cell protein extract at various stages of purification. *Lane 1* standard protein molecular weight markers (lysozyme, 14.4 kDa; *β*-lactoglobulin, 18.4 kDa; REase Bsp98 l, 25.0 kDa; lactate dehydrogenase, 35.0 kDa; ovalbumin, 45.0 kDa; bovine serum albumin, 66.2 kDa; *β*-galactosidase, 116.0 kDa). *Lane 2* crude cell-free extract. *Lane 3* ammonium sulfate fractionation. *Lane 4* Q-Sepharose column fraction. *Lane 5* phenyl-Sepharose column fraction

The apparent molecular mass of the purified nitrile hydratase was determined by gel filtration chromatography on a Sephacryl S200 column that had been pre-calibrated with the standard protein molecular weight markers. The molecular weight of the native enzyme was 65 kDa. For nitrile hydratase of *Rhodococcus equi* A4, an approximate molecular weight of 74 kDa has been reported (Přepechalová et al., 2001). The purified enzyme recovered from the gel filtration column was concentrated and used to determine the molecular weights of the subunits α and β. The silver stained denaturing SDS-PAGE gel for the purified enzyme is shown in Fig. 1. The purified enzyme exhibited two distinct bands with molecular weights of approximately 29 and 30 kDa (Fig. 1, lane 5), confirming the presence of two subunits. The α-subunit of nitrile hydratase of *R. rhodochrous* has been previously reported to be lighter in weight than the β-subunit (Nagasawa et al. 1991; Prasad et al. 2009) therefore, by analogy, the subunit with a molecular weight of around 29 kDa was assumed to be the α, and the other to be the β.

#### Temperature and pH optima

**Fig. 2:**
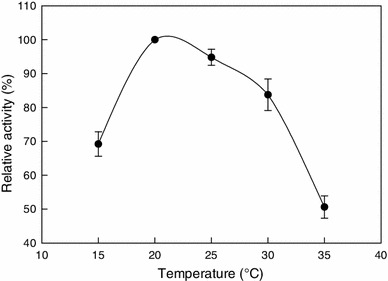
Activity–temperature profile of nitrile hydratase measured at pH 7.0 (20 mM phosphate buffer)

**Fig. 3:**
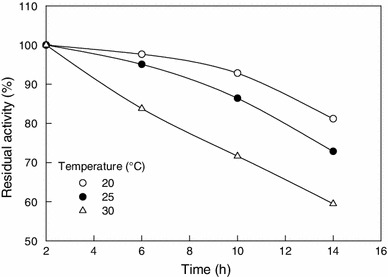
Residual activity of nitrile hydratase versus time at different incubation temperatures. The pH was 7.0

The effect of temperature (Cramp et al. 1997; Nagasawa et al. 1986; Prasad et al. 2009) and pH (Přepechalová et al. 2001; Hjort et al. 1990) on nitrile hydratase activity from various microbial sources has also been reported in literature. The relative activity of nitrile hydratase at various incubation temperatures is shown in Fig. 2. The temperature for peak activity was 20 °C (Fig. 2), but the temperature range for maximum activity was relatively broad at 20–25 °C (Fig. 2). At temperatures of >30 °C, the activity was reduced likely, because the elevated temperature adversely affected the stability of the enzyme. This might be due to thermal deactivation of the protein molecules. The thermostability of the purified enzyme was measured at various incubation temperatures (Fig. 3). The enzyme was clearly prone to increasing denaturation with the increasing temperature in the range of 25–30 °C. The relevant data are shown in Table 2. The thermal deactivation constant (*k*_d_, h^−1^) of the enzyme at various temperatures was estimated from the slope of a semilog plot of the enzyme activity versus time. The half-life (*t*_1/2_, h) of the enzyme was then estimated using the following equation:

**Table 2:** Thermal deactivation constant and half-life of nitrile hydratase at various temperatures (pH 7.0, 20 mM phosphate buffer)

Temperature (°C)	Thermal deactivation constant, *k*_d_ (h^−1^)	Half-life, *t*_1/2_ (h)
20	0.018	38.5
25	0.027	25.7
30	0.041	16.9

1

The half-lives of ferric type nitrile hydratases have been reported as 2.6 h and 15 min at 20 and 30 °C, respectively (Nagamune et al. 1990). Generally, cobalt-type nitrile hydratases are relatively more thermostable than the ferric type nitrile hydratase. The reported half-life of nitrile hydratase from *Bacillus pallidus* Dac521 at 30 °C was 40 h (Cramp et al. 1997). Half-life of nitrile hydratase from *R. rhodochrous* PA-34 was reported to be 2 h at 40 °C and 0.5 h at 50 °C (Prasad et al. 2009). The nitrile hydratase from *Brevibacterium* R-312 had half-lives of 240 and 48 h at 10 and 25 °C, respectively (Nagasawa et al. 1986).

**Fig. 4:**
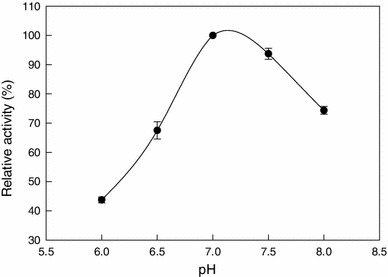
The activity–pH profile for nitrile hydratase at 20 °C

**Fig. 5:**
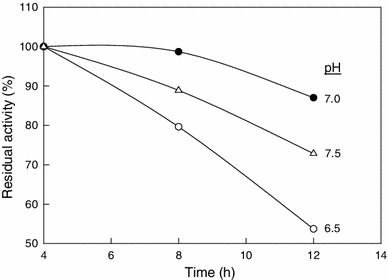
Residual activity of nitrile hydratase versus time at various values of the incubation pH. The temperature was 20 °C

pH is one of the most important factors influencing the side groups of the amino acid dissociation in the protein structure. The optimum pH for nitrile hydratase activity was determined by measuring the relative enzyme activity in buffers of different pH values. The optimum pH for the nitrile hydratase activity was 7.0, but the enzyme remained fairly active up to a pH of 7.5 (Fig. 4). The enzyme was also most stable at a pH of 7.0 (Fig. 5). Both higher and lower pH values reduced stability (Fig. 5).

The half-life (*t*_1/2_) of the nitrile hydratase at different pH values was estimated from the slopes of the semilog plots of residual activity versus time, as explained above. The relevant data are shown in Table 3.

**Table 3:** Half-life of nitrile hydratase at 20 °C and different pH values

pH	Half-life, *t*_1/2_ (h)
6.5	12
7.0	38
7.5	22

#### Substrate specificity

The substrate activity spectrum of nitrile hydratases from different sources can be quite different (Přepechalová et al. 2001; Bui et al. 1984; Asano et al. 1982; Nagasawa et al. 1987, 1986; Hjort et al. 1990). The activity of purified nitrile hydratase toward various nitriles (Table 4) was examined. The conversion of 3-cyanopyridine was taken to be 100 %. All other relative activity data (Table 4) are relative to the conversion of 3-cyanopyridine.

**Table 4:** Substrate specificity of nitrile hydratase from *R. erythropolis*

Type of nitrile	Structure	Relative activity^a^ (%)
*Aliphatic nitrile*		
Acetonitrile		54
Isobutyronitrile		110
*Aromatic nitrile*		
Benzonitrile		105
3-Aminobenzonitrile		56
4-Cyanopyridine		104
3-Cyanopyridine		100
Pyrazinonitrile		94
*Dinitrile*		
Succinonitrile		0
Adiponitrile		0

^a^Measured at pH 7.0 (20 mM phosphate buffer), 20 °C, after 30 min. The initial substrate concentration was always 2 mM

The enzyme displayed good activity toward aromatic nitriles, such as benzonitrile, 4-cyanopridine, 3-cyanopyridine and pyrazinonitrile (Table 4), but was less active on 3-aminobenzonitrile. The enzyme was quite active on the aliphatic nitrile, i.e., isobutyronitrile, but less on acetonitrile. The enzyme was completely inactive on aliphatic dinitriles (Table 4). The *meta* substitution of benzonitrile with an amino group reduced activity (Table 4), possibly as a consequence of the electron-donating effect of the substituent. The position of the substituent on the aromatic ring is known to affect activity as observed for nitrile hydratase of *Rhodococcus equi* A4 (Přepechalová et al. 2001).

#### Effect of metal ions and inhibitors

It is evident from Table 5 that the presence of various metal ions enhanced the enzyme activity. Cobalt(II) had the strongest enhancing effect. The metal ion chelating agent EDTA had a strong inhibitory effect, presumably because it bound the Co(III) at the active site of the enzyme. Urea was found to be a weak inhibitor of the enzyme (Table 5). Effect of metal ions on nitrile hydratase activity has been widely reported in literature (Maier-Greiner et al. 1991; Cramp et al. 1997; Wieser et al. 1998).

**Table 5:** Effect of metal ions and inhibitors on the nitrile hydratase activity

Metal ion or inhibitor	Relative activity^a^ (%)
None	100
CoCl_2_	215
FeCl_3_	174
FeSO_4_	183
NaCl	194
Urea	91
EDTA	34
2-Mercaptoethanol	68

^a^Measured in phosphate buffer (pH 7.0, 20 mM) at 20 °C, after 30 min of reaction using 3-cyanopyridine with various metal ions at an initial concentration of 2 mM

#### Kinetic constants

**Fig. 6:**
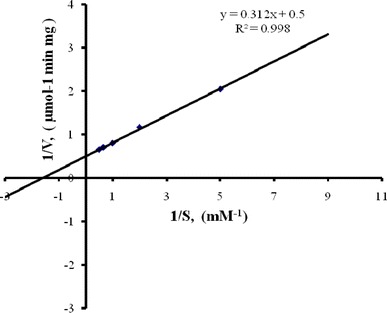
A Lineweaver–Burk plot for biotransformation of 3-cyanopyridine to nicotinamide using nitrile hydratase. The reciprocal of the initial reaction rate (*v*) is plotted against the reciprocal of the initial substrate concentration *S*. The data were measured at pH 7.0, 20 °C

The Michaelis–Menten constants *K*_m_ and *v*_max_ values were determined by plotting the initial reaction rate (*v*) versus the initial substrate concentration (*S*) in the form of a Lineweaver–Burk plot (Fig. 6). The substrate was 3-cyanopyridine and its concentration ranged from 0.2 to 2.0 mM. The reaction was carried out at pH 7.0 (20 mM phosphate buffer) and 25 °C. The *K*_m_ and *v*_max_ values were 0.624 mM and 5.12 μmol/min/mg, respectively. The *K*_m_ value obtained in this work was within the range reported for nitrile hydratases from other microorganisms (Wieser et al. 1998; Mayaux et al. 1990; Watanabe et al. 1987; Pereira et al. 1998; Sachi and Lindsay 2007; Feng et al. 2008). The *v*_max_ value of nitrile hydratase of *Brevibacterium* R312 acting on 3-cyanopyridine was reported to be 5 μmol/min/mg (Mayaux et al. 1990) or only about 50 % of the value found in the present study.

### Biotransformation of nitriles using whole cells

The use of freely-suspended whole cells and alginate matrix immobilized whole cells of *R. erythropolis* was examined for biotransformation of nitriles because it eliminated the expense of recovering and purifying the nitrile hydratase enzyme from the cells. Biotransformation catalyzed by whole cells is discussed in the following sections.

#### Freely-suspended whole cells

Washed whole cells recovered from the fermentation broth were suspended in phosphate buffer (20 mM, pH 7.0) at a concentration of 190 mg/L. In different experiments, different nitriles were added to the reaction mixture to obtain an initial concentration of 30, 60 and 90 mM. The reaction mixture was continuously stirred (160 rpm) and incubated at 25 °C. The percent conversion for the various substrates after 24 and 48 h of the reaction is shown in Table 6. For all substrates, the conversion increased with time (Table 6), as expected. In all cases, the conversion at a given time was reduced with an increase in the initial concentration of the substrate (Table 6). For all the substrate tested, a conversion of ≥80 % could be achieved within 24 h starting with an initial substrate concentration of 30 mM (Table 6). An earlier study reported on the biotransformation of 3-cyanopyridine to nicotinamide using freely-suspended whole cells of *R. erythropolis* (Kamble and Banerjee 2008). A substrate conversion of 93 % was achieved under statistically optimized reaction conditions.

**Table 6:** Effect of nitrile type and concentration on percent conversion to the corresponding amide using free cells of *R. erythropolis*

Nitrile	Reaction time					
	24 h			48 h		
	30 mM	60 mM	90 mM	30 mM	60 mM	90 mM
Benzonitrile	95 ± 0.7	82 ± 2.1	77 ± 1.4	96 ± 1.4	91 ± 1.4	87 ± 2.1
4-Cyanopyridine	88 ± 1.4	77 ± 2.1	75 ± 1.4	94 ± 1.4	80 ± 1.4	76 ± 1.4
Pyrazinonitrile	80 ± 1.4	73 ± 1.4	65 ± 1.4	88 ± 1.4	77 ± 0.7	68 ± 1.4
Isobutyronitrile	83 ± 0.7	90 ± 0.7	79 ± 1.4	81 ± 0.7	88 ± 1.4	77 ± 2.1

#### Immobilized whole cells

##### Optimization of reaction parameters

Immobilization allows the cells to be reused relatively easily and often improves the half-life of the biocatalyst (Adinarayana et al. 2005; Kaul et al. 2006; Vejvoda et al. 2006; Fatima et al. 2007; Ahmed 2008; Bhattacharyya et al. 2010). Furthermore, the immobilized cells are relatively easily retained in a continuous flow biotransformation reactor. This was the rationale of evaluation of immobilized cells for nitrile biotransformations in this study.

##### Alginate concentration

**Fig. 7:**
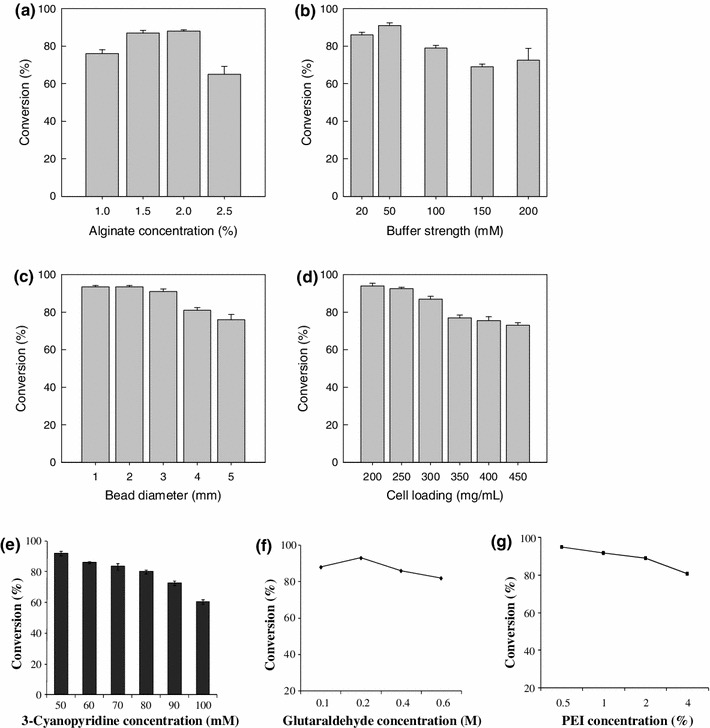
Conversion of 3-cyanopyridine at 24 h (25 °C, pH 7.0) using **a** different alginate concentrations in the beads, **b** different concentrations of the Tris–HCl buffer in the reaction medium, **c** different bead sizes (2 % alginate in beads), **d** different cell loadings in the beads, **e** different substrate (3-cyanopyridine) concentrations, **f** different glutaraldehyde concentration, **g** different polyethyleneimine concentration

Different concentrations (1.0–2.5 %, g/100 mL) of alginate were tested for preparing the gel matrix for immobilizing the cells. In all cases, the bead diameter was 2 mm and the cell loading in the gel matrix was 190 mg/mL. The beads were tested for the conversion of 3-caynopyridine to nicotinamide at 25 °C in pH 7.0 buffer (50 mM Tris–HCl) for 24 h. Beads made with 1.5 and 2 % alginate concentration achieved the highest conversion of 86 % (Fig. 7a). The conversion was lower at an alginate concentration of <1.5 %, possibly because of a reduced entrapment of the cells in the softer beads. The conversion was also reduced if the alginate concentration exceeded 2 % (Fig. 7a) likely, because of increased diffusional limitation within the beads. A decreased mass transfer and conversion have been previously reported for beads made with a high concentration of alginate (Srinivasulu et al. 2003). In view of the results in Fig. 7a, an alginate concentration of 2 % was used in all future experiments.

##### Selection of buffer strength

Different strengths (20, 50, 100, 150 and 200 mM) of Tris–HCl buffer (pH 7.0) were evaluated for the biotransformation with immobilized cells. The biocatalyst beads and the reaction conditions were as described in the previous section. The results are shown in Fig. 7b. A buffer concentration of 50 mM (Tris–HCl buffer, pH 7.0) provided a conversion of 90 % and was therefore selected for all future work.

##### Bead size

Bead size affects mass transfer within the bead and the fraction of the cells in the bead volume that can be used effectively for a reaction. Therefore, the effect of bead size on the reaction was evaluated. Beads of different sizes (1–5 mm) were generated using syringes with different orifice sizes. The reaction conditions were: 2 % alginate concentration in the beads, 50 mM (Tris–HCl buffer, pH 7.0), a cell loading of 190 mg/mL, 25 °C, 3-caynopyridine at an initial concentration of 22 mM. The conversion after 24 h was the highest with beads having a diameter of 1–3 mm (Fig. 7c). As the bead diameter reduced, the surface to volume ratio increased and therefore the mass transfer per unit volume improved. This explained the higher conversion with the smaller beads. A bead size of 3 mm was selected for all further work, as these relatively large beads were easier to handle and physically more stable than the smaller beads.

##### Substrate concentration

The effect of the initial substrate concentration (50–100 mM, 3-caynopyridine) on the biotransformation reaction was tested using 3-mm beads. The other reaction conditions were as in the previous section. At 50 mM substrate concentration, the conversion exceeded 90 %, but higher concentrations of the substrate reduced conversion (Fig. 7e). The decrease in the conversion at high substrate level may be due to substrate inhibition of the enzyme. Therefore, a 50 mM substrate concentration was selected for all further work.

##### Cell mass concentration

Using beads of 3 mm diameter, the effect of cell loading (200, 250, 300, 350, 400 and 450 mg/mL) in the gel matrix, on the conversion of 3-cyanopyridine to nicotinamide was assessed. The reaction conditions were: 25 °C, pH 7.0 (50 mM Tris–HCl buffer), 2 % alginate in the gel matrix and 50 mM initial substrate concentration. The cell loadings of 200 and 250 mg/mL provided good conversion (95 %) of 3-cyanopyridine (Fig. 7d). Higher cell loading likely impeded mass transfer of the substrate and product within the bead and were therefore less effective. Similar effects of cell loading in gel beads have been previously reported (Lee et al. 1995). In view of the results (Fig. 7d), a cell loading of 200 mg/mL was selected for all further work.

##### Effect of bead hardening treatments

To increase the stability of the beads and their potential for reuse, the surface crosslinking with glutaraldehyde and polyethyleneimine have been reported in literature (Hann et al. 2002). In this work the beads with the immobilized cells were cross-linked by suspending in a mixture of glutaraldehyde (0.2 M) and 0.5 % (g/100 mL) polyethyleneimine for 1 h at room temperature. Afterwards, the beads were repeatedly washed with deionized water and used for the hydration of 3-cyanopyridine. The reaction conditions were: 25 °C, pH 7.0 (50 mM Tris–HCl buffer), 2 % alginate in the gel matrix, 200 mg/mL cell loading and 50 mM initial substrate concentration. A substrate conversion of >95 % could be achieved with the cross-linked immobilized beads (Fig. 7f, g).

Using the cross-linked beads, the biotransformation of different substrates (Table 7) at different initial concentrations (60, 100 and 150 mM) was tested in separate experiments. The reaction conditions were: 25 °C, pH 7.0 (50 mM Tris–HCl buffer), 2 % alginate in the gel matrix and a cell loading in the beads of 200 mg/mL. For the aromatic nitriles (i.e., benzonitrile, 4-cyanopyridine, pyrazinonitrile), the conversion was generally good up to a substrate concentration of 100 mM (Table 7). A further increase in the initial concentration of the substrate reduced conversion. For the non-aromatic isobutyronitrile, the conversion was generally higher than for the aromatic substrates (Table 7). A comparison of Tables 6 and 7 shows that at a given concentration of the substrate the immobilized cells were more effective than the freely-suspended cells for these biotransformations. Unlike the freely-suspended cells, the immobilized cells could of course be reused relatively easily. Immobilization in beads improved the cells’ tolerance toward the substrates.

**Table 7:** Effect of nitrile type and concentration on percent conversion to the corresponding amide using immobilized cells of *R. erythropolis*

Nitrile	Reaction time					
	24 h			48 h		
	60 mM	100 mM	150 mM	60 mM	100 mM	150 mM
Benzonitrile	89 ± 1.4	81 ± 1.4	80 ± 1.4	97 ± 0.7	88 ± 1.4	84 ± 0.7
4-Cyanopyridine	79 ± 0.7	66 ± 0.7	62 ± 0.7	94 ± 1.4	88 ± 0.7	80 ± 1.4
Pyrazinonitrile	81 ± 2.1	66 ± 1.4	61 ± 1.4	89 ± 1.4	79 ± 1.4	75 ± 1.4
Isobutyronitrile	98 ± 1.4	95 ± 1.4	88 ± 0.7	96 ± 1.4	92 ± 1.4	73 ± 0.7

#### Use of packed bed reactor for the nitrile conversion

A vertically mounted jacketed packed bead reactor (length 18 cm, diameter 2.6 cm, working volume 96 mL) was used in these experiments. The bed was packed with the alginate beads (3 mm diameter, 2 % alginate in beads, 200 mg/mL cell loading in the gel matrix). The substrate was fed at the bottom of the reactor using a peristaltic pump. The substrate concentration in the feed was always 100 mM and the substrate was dissolved in buffer (pH 7.0, 50 mM Tris–HCl). The reactor was maintained at 25 °C. The effect of the substrate flow rate on conversion and the reusability of the immobilized beads were evaluated.

##### Effect of flow rate on conversion

**Fig. 8:**
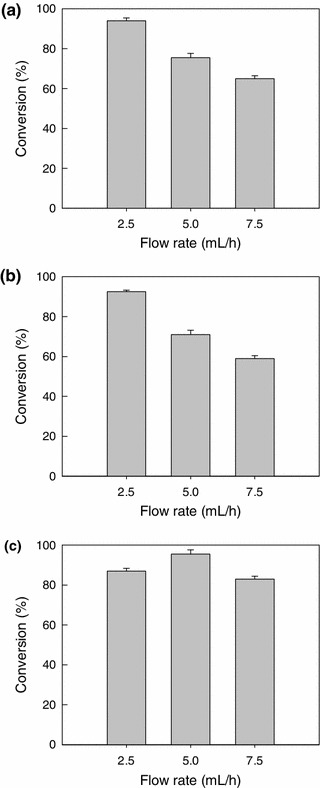
Effect of substrate flow rate in the packed bed on the conversion of: **a** benzonitrile, **b** 4-cyanopyridine and **c** isobutyronitrile

The substrate flow rate (2.5, 5.0 and 7.5 mL/h) affected the conversion as shown in Fig. 8 for the different nitriles. For the flow rate values of 2.5, 5.0 and 7.5 mL/h, the residence times in the reactor were 38, 19.4 and 12.8 h, respectively. Conversion generally decreased with the increase in substrate flow rate, as the residence time, or the time available for the reaction, reduced (Chang et al. 2009). A flow rate of 2.5 mL/h afforded good conversion of benzonitrile and 4-cyanopyridine (Fig. 8).

For isobutyronitrile, the flow rate for optimal conversion was 5 mL/h. Lower and higher flow rates reduced conversion (Fig. 8). A reduced conversion as a consequence of an increased flow rate is of course easily understood in terms of a reduced amount of time available for the reaction. The increased conversion of isobutyronitrile with an increase in flow rate from 2.5 to 5.0 mL/h at first seems unusual. In shake flask batch reactors (Table 7), the conversion of this substrate was actually lower at 48 h compared to the conversion at 24 h. Clearly, therefore, an excessively long reaction time actually reduced conversion of this substrate. This may be a consequence of the equilibrium kinetics of the enzyme-catalyzed reactions.

The packed bed reactor had a superior performance compared to the batch reactors (Table 7). For example, for benzonitrile and 4-cyanopyridine, at a residence time of 38 h (i.e., a flow rate of 2.5 mL/h) in the packed reactor, the conversion was >90 % (Fig. 8). For the same substrates at 48 h (Table 7) starting with a substrate concentration of 100 mM as in the packed bed, the conversion values were much lower: 81 % for benzonitrile and 66 % for 4-cyanopyridine (Table 7). Similarly, using isobutyronitrile the conversion in the packed bed was 95 % at a residence time of ~19 h (i.e., a flow rate of 5.0 mL/h; Fig. 8), but in the batch rector the same conversion was achieved in 24 h (Table 7).

##### Reusability of catalysts

**Fig. 9:**
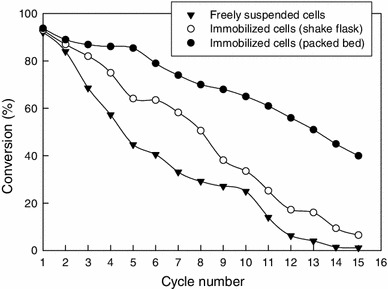
Effect of reuse cycle number on the conversion achieved by different preparations of the biocatalyst

The reusability of the freely-suspended cells in shake flasks, the immobilized whole cells in shake flasks and the immobilized cells in the packed bed reactor is compared in Fig. 9. The immobilized cells used in the packed bed had been subjected to the earlier mentioned cross-linking treatment. The data in Fig. 9 are for the conversion of 3-cyanopyridine (50 mM) at 25 °C, pH 7 (50 mM Tris–HCl buffer). The immobilized beads were 3 mm in diameter and had been prepared using an alginate concentration of 2 %. The cell loading in the beads was 200 mg/L. After each use cycle, the cells and the beads were recovered, washed and reused for the next cycle. With all the catalyst systems, the conversion declined with increasing number of use cycles (Fig. 9). The freely-suspended cells lost activity most rapidly. The immobilized cells in shake flasks were relatively more stable, but the cross-linked immobilized cells used in the packed bed were clearly the most stable (Fig. 9). The cross-linked immobilized cells in the packed bed could be used for at least six cycles while continuing to achieve a substrate conversion of ~85 %.

## Concluding remarks

The intracellular nitrile hydratase of *R. erythropolis* was purified and characterized. The pH and temperature optima for the enzyme activity were 7.0 and 20 °C, respectively. The enzyme-containing freely-suspended whole cells and the cells immobilized in alginate beads were highly effective in converting various nitriles to amides, but the immobilized cells could be reused. The use of the immobilized cells of *R. erythropolis* MTCC 1526 in packed beds offers an alternative to the traditional chemical processes for converting nitriles to amides.

## References

[CR1] Adinarayana K, Jyothi B, Ellaiah P (2005). Production of alkaline protease with immobilized cells of *B. subtilis* PE-11 in various matrices by entrapment technique. AAPS PharmSciTech.

[CR2] Ahmed SA (2008). Invertase production by *Bacillus macerans* immobilized on calcium alginate beads. J Appl Sci Res.

[CR3] Asano Y, Fujishiro K, Tani Y, Yamada H (1982). Aliphatic nitrile hydratase from *Arthrobacter* sp. J-1: purification and characterization. Agric Biol Chem.

[CR4] Bhattacharyya MS, Singh A, Banerjee UC (2010). Immobilization of intracellular carbonyl reductase from *Geotrichum candidum* for the stereoselective reduction of 1-naphthyl ketone. Bioresour Technol.

[CR5] Bradford MM (1976). A rapid and sensitive method for quantisation of microgram amount of protein using the principle of protein-dye binding. Anal Biochem.

[CR6] Brady D, Beeton A, Zeevaart J, Kgaje C, van Rantwijk F, Sheldon RA (2004). Characterization of nitrilase and nitrile hydratase biocatalytic systems. ApplMicrobiol Biotechnol.

[CR45] Bui K, Fradet H, Arnaud A, Galzy P (1984). A nitrile hydratase with a wide substrate spectrum produced by a *Brevibacterium* sp. J Gen Microbiol.

[CR7] Cantarella M, Cantarella L, Gallifuoco A, Spera A (2006). Use of a UF-membrane reactor for controlling selectively the nitrile hydratase-amidase system in Microbacterium imperial CBS 498–74 resting cells. Case study: benzonitrile conversion. Enzyme Microb Technol.

[CR8] Chang C, Chen J-H, Chang CJ, Wu T-T, Shieh CJ (2009). Optimization of lipase-catalyzed biodiesel by isopropanolysis in a continuous packed-bed reactor using response surface methodology. New Biotechnol.

[CR9] Clifford KH, Geary PJ, Pryce RJ (1993) Process for the preparation of difluorobenzamide.US Patent 5,206,158

[CR10] Cramp RA, Gilmour M, Cowan DA (1997). Novel thermophilic bacteria producing nitrile-degrading enzymes. Microbiol.

[CR11] Dráber P (1991). Quantification of proteins in sample buffer for sodium dodecyl sulfate polyacrylamide gel electrophoresis using colloidal silver. Electrophor.

[CR12] Fatima Y, Kansal H, Banerjee UC (2007). Enantioselective reduction of aryl ketones using immobilized cells of *Candida viswanathii*. Process Biochem.

[CR13] Feng YS, Chen PC, Wen FS, Hsiao WY, Lee CM (2008). Nitrile hydratase from *Mesorhizobium* sp. F28 and its potential for nitrile biotransformation. Process Biochem.

[CR15] Hann EC, Sigmund AE, Hennessey SM, Gavagan JE, Short DR, Ben-Bassat A, Chauhan S, Fallon RD, Payne MS, DiCosimo R (2002). Optimization of an immobilized-cell biocatalyst for production of 4-cyanopentanoic acid. Org Proc Res Dev.

[CR16] Hjort CM, Godtfredsen SE, Emborg C (1990). Isolation and characterization of a nitrile hydratase from a *Rhodococcus* sp. J Chem Technol Biotechnol.

[CR17] Kamble A, Banerjee UC (2008). Optimization of crucial reaction conditions for the production of nicotinamide by nitrile hydratase using response surface methodology. Appl Biochem Biotechnol.

[CR18] Kaul P, Banerjee A, Banerjee UC (2006). Stereoselective nitrile hydrolysis by immobilized whole-cell biocatalyst. Biomacromolecules.

[CR19] Kobayashi M, Shimizu S (2000). Nitrile hydrolyases. Curr Opin Chem Biol.

[CR20] Laemmli UK (1970). Cleavage of structural proteins during the assembly of the head of bacteriophage T4. Nature.

[CR21] Lee W-C, Lee R-Y, Ruaan R-C (1995). Effect of cell concentration on the kinetics of whole-cell enzyme entrapped within calcium alginate. Biotechnol Prog.

[CR22] Lin S-H, Chong ZZ, Maiese K (2001). Nicotinamide: a nutritional supplement that provides protection against neuronal and vascular injury. J Med Food.

[CR23] Maier-Greiner UH, Obermaier-Skrobranek BMM, Estermaier LM, Kammerloher W, Freund C, Wulfing C, Burkert UI, Matern DH, Breuer M, Eulitz M (1991). Isolation and properties of a nitrile hydratase from the soil fungus *Myrothecium verrucaria* that is highly specific for the fertilizer cyanamide and cloning of its gene. PNAS.

[CR24] Mascharak PK (2002). Structural and functional models of nitrile hydratase. Coord Chem Rev.

[CR25] Mayaux J, Cerbelaud E, Soubrier F, Faucher D, Petre D (1990). Purification, cloning, and primary structure of an enantiomer selective amidase from *Brevibacterium* sp. R312: structural evidence for genetic coupling with nitrile hydratase. J Bacteriol.

[CR26] Mylerova V, Martinkova L (2003). Synthetic applications of nitrile-converting enzymes. Curr Org Chem.

[CR29] Nagamune T, Kurata H, Hirata M, Honda J, Hirata A, Endo I (1990) Photosensitive phenomena of nitrile hydratase of *Rhodococcus* sp. N-771. Photochem Photobiol 51:87–90

[CR27] Nagasawa T, Ryuno K, Yamada H (1986). Nitrile hydratase of *Brevibacterium* R312: purification and characterization. Biochem Biophys Res Commun.

[CR28] Nagasawa T, Nanba H, Ryuno K, Takeuchi K, Yamada H (1987). Nitrile hydratase of *Pseudomonas chlororaphis* B23 purification and characterization. Eur J Biochem.

[CR30] Nagasawa T, Takeuchi K, Yamada H (1991). Characterization of a new cobalt-containing nitrile hydratase purified from urea-induced cells of *R. rhodochrous* J1. Eur J Biochem.

[CR31] Nigam VK, Khandelwal AK, Gothwal RK, Mohan MK, Choudhury B, Vidyarthi AS, Ghosh P (2009). Nitrilase-catalysed conversion of acrylonitrile by free and immobilized cells of *Streptomyces* sp. J Biosci.

[CR32] Payne MS, Wu S, Fallon RD, Tudor G (1997). A stereoselective cobalt-containing nitrile hydratase. Biochem.

[CR33] Pereira RA, Graham D, Rainey FA, Cowan DA (1998). A novel thermostable nitrile hydratase. Extremophiles.

[CR34] Prasad S, Raj J, Bhalla TC (2009). Purification of a hyperactive nitrile hydratase from resting cells of *Rhodococcus rhodochrous* PA-34. Indian J Microbiol.

[CR35] Přepechalová I, Martínková L, Stolz A, Ovesná M, Bezouška K, Kopecký J, Křen V (2001). Purification and characterization of the enantioselective nitrile hydratase from *Rhodococcus equi* A4. Appl Microbiol Biotechnol.

[CR36] Raj J, Prasad S, Bhalla TC (2006). *Rhodococcus rhodochrous* PA34: a potential biocatalyst for acrylamide synthesis. Process Biochem.

[CR37] Ray SC, Nandi LN, Singh B, Prasad H, Maharaj S, Sarkar PK, Dutta P, Roy SK, Yadav SN, Bandyopadhyay AK (2004) Process for the synthesis of isonicotinic acid hydrazide.US Patent 6,734,309

[CR38] Sachi O, Lindsay D (2007). Purification and characterization of a novel nitrile hydratase from *Rhodococcus* sp. RHA1. Mol Microbiol.

[CR39] Srinivasulu B, Adinarayana K, Ellaiah P (2003). Investigations on neomycin production with immobilized cells of *Streptomyces marinensis* Nuv-5 in calcium alginate matrix. AAPS PharmSciTech.

[CR40] Sugiura Y, Kuwahara J, Nagasawa T, Yamada H (1987). Nitrile hydratase: the first non-heme iron enzyme with a typical low-spin Fe(III)-active center. J Am Chem Soc.

[CR41] Tanaka K, Kimura K (1972). Process for preparing amino acids from hydrocarbons. US Patent.

[CR42] Vejvoda V, Kaplan O, Kubáč D, Křen V, Martinková L (2006). Immobilization of fungal nitrilase and bacterial amidase—two enzymes working in accord. BiocatalBiotrans.

[CR43] Watanabe I, Satoh Y, Enomoto K, Seki S, Sakashita K (1987). Optimal conditions for cultivation of *Rhodococcus* sp. N-774 and for conversion of acrylonitrile to acrylamide by resting cells. AgricBiolChem.

[CR44] Wieser M, Takeuchi K, Wada Y, Yamada H, Nagasawa T (1998). Low-molecular-mass nitrile hydratase from *Rhodococcus rhodochrous* J1: purification, substrate specificity and comparison with the analogous high-molecular-mass enzyme. FEMS Microbiol Lett.

[CR46] Yamada H, Shimizu S, Kobayashi M (2001). Hydratase involved in nitrile conversion: screening, characterization and application. The Chem Record.

